# Antitumor Effects of Berberine on Gliomas via Inactivation of Caspase-1-Mediated IL-1β and IL-18 Release

**DOI:** 10.3389/fonc.2019.00364

**Published:** 2019-05-14

**Authors:** Lei Tong, Chuncheng Xie, Yafen Wei, Yunyue Qu, Hongsheng Liang, Yiwei Zhang, Tianye Xu, Xin Qian, Huijia Qiu, Haoyu Deng

**Affiliations:** ^1^Department of Microbiology, Harbin Medical University, Harbin, China; ^2^Department of Neurosurgery, The First Affiliated Hospital of Harbin Medical University, Harbin, China; ^3^Department of Neurology, The Provincal Hospital of Heilongjiang Province, Harbin, China; ^4^Department of Vascular Surgery, RenJi Hospital, Shanghai Jiaotong University School of Medicine, Shanghai, China; ^5^Department of Neurosurgery, Shanghai Tenth People's Hospital, Tongji University School of Medicine, Shanghai, China

**Keywords:** gliomas, berberine, EMT, ERK1/2, IL-1β, IL-18

## Abstract

Gliomas arise in the glial cells of the brain or spine and are the most prevalent and devastating type of brain tumors. Studies of tumor immunology have established the importance of the tumor micro-environment as a driver of oncogenesis. Inflammatory mediators such as IL-1β and IL-18 released by monocytes regulate transcriptional networks that are required for malignant cell growth. Berberine is a natural botanical alkaloid that is widely found in the Berberis species. Although it has been widely used as an anti-diarrheal treatment in North America for several decades, our study is the first to investigate berberine as an anti-tumor agent in glioma cells. In this study, we demonstrate that berberine significantly inhibits inflammatory cytokine Caspase-1 activation via ERK1/2 signaling and subsequent production of IL-1β and IL-18 by glioma cells. Moreover, we found that berberine treatment led to decreased motility and subsequently cell death in U251 and U87 cells. In addition, our study is the first to indicate that berberine can reverse the process of epithelial-mesenchymal transition, a marker of tumor invasion. Taken together, our work supports berberine as a putative anti-tumor agent targeting glioma cells.

## Introduction

Gliomas are one of the most prevalent primary intracranial tumors, representing about 81% of malignant brain tumors. Although relatively rare, it usually causes significant mortality and morbidity (1). So far, gliomas remain incurable, despite many attempted treatment approaches (2). Concomitant loss of chromosomes 1p and 19q is one of the best studied molecular alterations in gliomas and is strongly associated with oligodendroglia morphology and mortality (3). In addition, high mutation rates of p53 in gliomas is reported to be responsible for the initiation of rapid glioma growth, which has led researchers to examine this alteration in association with glioma onset (4). Bevacizumab, a targeted therapeutic for gliomas, still does not improve overall survival (OS) in either recurrent gliomas or newly-diagnosed gliomas. Thus, novel, effective, targeted therapies for gliomas are in urgent need (5).

Inflammation is a key component of the glioma microenvironment. Neuroinflammatory cytokines such as interleukin-1 (IL-1) secreted by glioma cells are known to contribute to tumor initiation and early progression (6). The inflammatory response and cancer are highly linked by specific intrinsic pathways, whereby genetic alterations that cause cancer orchestrate the proinflammatory microenvironment, and IL-1 has been associated in this process. For example, IL-1α is a downstream effector of Ras activation and is essential for Nuclear factor-[kappa]B (NF-κB)-regulated gene activation, which includes cytokines and chemokines required for the establishment of a favorable microenvironment for oncogenesis (7). A recent phase 2 clinical trial of a recombinant IL-1R antagonist against multiple myeloma demonstrated a favorable safety profile and improved the disease-free rate, providing evidence that anti-IL-1 therapy is an effective therapeutic strategy against cancer (8).

Berberine is a compound isolated from a Chinese herb, which has a wide spectrum of pharmacological effects as an anti-cancer, anti-inflammatory, and neuroprotective treatment (9). The immunomodulatory effect of berberine that underlies its neuroprotective activities has been well-documented in several preclinical and clinical studies (10). For example, the therapeutic effects of berberine on host immune cells such as lymphocytes, leukocytes, astrocytes, and microglial cells in neurological, inflammatory and autoimmune disorders have been well**-**explored (11). Recently, studies have shown berberine can exert antitumor activities both *in vitro* and *in vivo* through distinct mechanisms including transcriptional regulation of oncogenes and carcinogenesis-related gene expression, modulation of reactive oxygen species production, mitochondrial transmembrane potential, and NF-κB activation (12). Moreover, berberine was found to inhibit tumor growth through cell cycle arrest and apoptosis in various types of tumors, including leukemia, liver cancer, gastric cancer, colon cancer, and breast cancer (13).

However, it remains unclear if anti-inflammatory effect of berberine translates into an anti-tumor effect in glioma cells. In this study, we investigated the effects of berberine on glioma cells and further evaluated the underlying mechanisms of berberine-induced anti-tumor activity.

## Materials and Methods

### Collection of Glioma and Non-tumorous Human Brain Tissues

Human glioma tissues and non-tumorous brain tissues were obtained by surgical removal at the first affiliated hospital of Harbin Medical University. Each glioma sample was graded according to the guideline released by WHO. The study was approved by the ethics committee of Harbin Medical University and written informed consent was obtained from each patient.

### Immunohistochemical Analysis

Paraffin sections were heated at 60°C, deparaffinized in xylene, rehydrated in graded ethanol and microwaved for antigen retrieval. Slides were incubated with primary antibodies against caspase-1 (#3866; Cell Signaling Technology, Beverly, MA, USA), IL-18 (10663-1-AP, Proteintech, Wuhan, China), and IL-1β (16806-1-AP, Proteintech, Wuhan, China) at 4°C overnight. Slides were processed for incubation with secondary antibodies for 2 h at room temperature and stained with diaminobenzidine.

### Cell Culture and Drug Treatment

Human U87 and U251 cell lines and oligodendrocytes were purchased from American Type Culture Collection. The cells were cultured in DMEM (11965118; Invitrogen, Shanghai, China) supplemented with 10% fetal bovine serum (04-001-1; Biological industries, Beit-Haemek, Israel). For drug treatments, U87 and U251 cells were treated with a specific caspase-1 inhibitor N-Ac-Tyr-Val-Ala-Asp-CMK (Ac-YYAD-CMK) (10014; Cayman Chemical, Ann Arbor, MI, USA) and U0216, a MEK1 and MEK2 inhibitor (U120; Sigma-Aldrich, St. Louis, USA), respectively, at indicated dosage.

### MTT Assay

Cell viability was determined by MTT assays according to the manufacturer's instructions. Briefly, cells (2 × 10^4^ cells/well) treated with either berberine or Ac-YVAD-MK were seeded in a 96-well plate. Twenty microliter of MTT solution (88417; Sigma, St. Louis, MO, USA) was added to each well and incubated at 37°C for 4 h followed by dimethyl sulfoxide (DMSO) incubation to dissolve formazine granules. The absorbance at 490 nm was measured using a microplate reader.

### Wound Healing Assay

U87 and U251 cells were incubated in a 6-well plate at a confluence of 90%. The cell monolayer was scratched in a straight line with a pipette tip. The wound area was quantified using ImagePro Plus 7.0 software (Media Cybernetics, Rockville, Maryland, USA), and the ratio of the healing area relative to the initial wound area was calculated. Quantification of bands was performed using the ImageJ program (National Institutes of Health, Bethesda, Maryland, USA). Three random fields of view were visualized and photographed using an inverted microscope.

### Immunofluorescence Staining

Cells growing on coverslips were rinsed with PBS for 3 × 5 min and then fixed with 4% paraformaldehyde for 30 min. Cells were permeabilized with 0.1% Triton-100 for 15 min followed by three washes with PBS. The coverslips were then blocked with 1% BSA in PBS for 30 min at 37°C and then incubated with primary antibody at a dilution of 1:100 at 4°C overnight. Cells were incubated with FITC-conjugated anti-rabbit IgG (H + L) antibodies. After three washes, the cells were incubated with 1 μg/ml DAPI in PBS for 5 min. The coverslips were observed using an Axiovert 200 (Zeiss) fluorescence microscope.

### Enzyme-Linked Immunosorbent Assay (ELISA)

Supernatant was collected for the measurement of IL-1β and IL-18 concentration using ELISA kits (EK0864 and EK0392; Boster, Wuhan, China) according to manufacturer's instructions. Briefly, the supernatant of cell cultures was collected and IL-1β and IL-18 were determined by ELISA. The optical density at 450 nm was determined using the microplate reader Epoch 2 (BioTek).

### Real-Time PCR Analysis

Total RNA was extracted using TRIzol reagent (15596018; Invitrogen, Carlsbad, CA, USA) from either brain tissues or U87 cells. First-strand cDNA was synthesized using PrimeScript reverse transcription kit (RR047Q; TaKaRa, Shiga, Japan) according to the manufacturer's instructions. Real-time PCR was carried out with a SYBR Green PCR Master Mix Kit (4309155; Applied Biosystems, CA, USA) and performed on 7500 FAST Real-Time PCR System (Applied Biosystems, Carlsbad, CA, USA). GAPDH was used as a housekeeping gene.

### Western Blot Analysis

Cells were washed with cold PBS and treated with RIPA buffer (89900; Thermo, Rockford, IL, USA) containing protease inhibitor cocktail (4693132001; Roche) and 1% phenylmethylsulfonyl fluoride PMSF (ST505; Beyotime, Shanghai, China). Proteins were quantified by Bradford assay and separated using a 12% SDS-PAGE gel prior to transferring onto a polyvinylidene difluoride (PVDF) membrane. Protein bands were visualized by enhanced chemiluminescence technique using SuperSignal West Pico chemiluminescent substrate (34577; Thermo, CA, USA). Densitometric quantification was performed using ImageJ v1.48 and the relative band intensity for each protein of interest was normalized against GAPDH. The primary antibodies used in this study included antibodies against GAPDH (60004-1-Ig; Proteintech, Wuhan, China), ACTIN (20536-1-AP; Proteintech), caspase-1 **(**#3866; Cell Signaling), α-catenin (#2131; Cell Signaling), β-catenin (#8480; Cell Signaling), α-SMA (#19245, Cell Signaling), vimentin (#5741; Cell Signaling). The goat anti-rabbit or anti-mouse horse radish peroxidase (HRP)-labeled antibodies were obtained from Proteintech.

### Statistical Analysis

Correlations between continuous variables were evaluated by Spearman correlation analysis. The results were expressed as mean ± standard error of mean (mean ± SEM) and analyzed with SPSS 13.0 software. Statistical significance between two individual groups were performed using unpaired Student's *t*-test. Statistical significance among multiple groups were performed using analysis of variance (ANOVA). A two-tailed *p* < 0.05 was considered as statistically significant. The survival distributions were described by the Kaplan-Meier survival curve and the log-rank test was used to test the statistical significance. Each experiment has been independently performed at least three times.

### Data Collections

A total of 1,510 cases were included in our study: Chinese Glioma Genome Atlas network (CGGA, *n* = 325, http://www.cgga.org.cn/), The Cancer Genome Atlas (TCGA) mRNA-seq data (*n* = 699, http://portal.gdc.cancer.gov/), and mRNA-microarray data the Repository for Molecular Brain Neoplasia (Georgetown Database of Cancer G-Doc, http://gdoc.georgetown.edu/gdoc/). Corresponding clinical information were downloaded from public databases, respectively.

## Results

### IL-1β and IL-18 Expression Is Associated With Clinical and Molecular Characteristics in Gliomas

**Figure 1:**
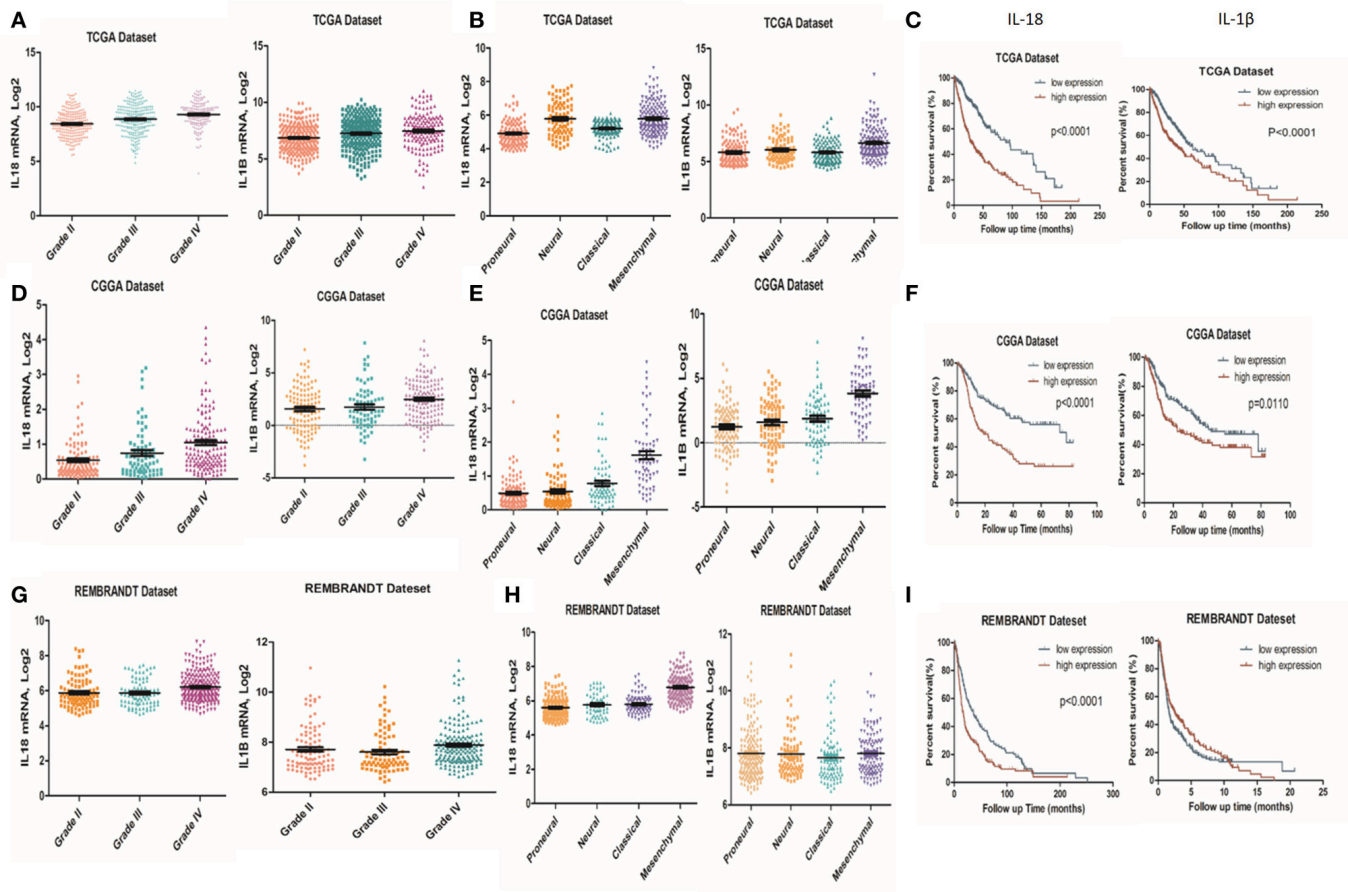
Associations of IL-1β and IL-18 expression with clinical and molecular characteristics in gliomas. IL-18 and IL-1β mRNA level was significantly increased in Grade IV gliomas as compared to Grade II. The results were obtained from TCGA **(A)**, CGGA **(D)** and REMBRANDT **(G)** cohorts, respectively. IL-18 and IL-1β mRNA levels were significantly increased in mesenchymal gliomas as compared to proneural gliomas. The results were obtained from TCGA **(B)**, CGGA **(E)**, and REMBRANDT **(H)** cohorts, respectively. Kaplan meier method was applied to compute overall median survival between patients with high expression of IL-18, IL-1β and low expression of IL-18, IL-1β. The data was collected from TCGA **(C)**, CGGA **(F)**, and REMBRANDT **(I)** cohorts, respectively.

It is well-established that inflammation within the glioma microenvironment plays a major role in determining the consequence of glioma progression, invasion, and resistance to therapeutic intervention (14). We first performed clinical data analysis to determine the association of IL-1β and IL-18 expression with clinical and molecular characteristics in gliomas. Due to the histopathological heterogeneity of gliomas, mRNA levels of IL-1β and IL-18 in gliomas were analyzed according to the WHO grade system and histology. In the TCGA, CGGA, and REMBRANDT datasets, we demonstrated that the expression of IL-1β and IL-18 were significantly increased in grade IV gliomas, compared to those in WHO grade II and grade III gliomas (Figures 1A,D,G). Further, IL-1β and IL-18 expression was significantly increased in mesenchymal gliomas than other types of gliomas (Figures 1B,E,H). Since IL-1β and IL-18 were positively associated with disease progression, we further investigated the prognostic values of IL-1β and IL-18 by Kaplan-Meier analysis. As demonstrated in Figures 1C,F,I, patients with increased expression of IL-1β and IL-18 had significantly shorter survival as compared to other patients. Taken together, these results indicate that increased expression of IL-1β and IL-18 is associated with poor prognostic outcomes for glioma patients.

### Expression of Caspase-1, IL-18, and IL-1β Is Significantly Upregulated in Gliomas Compared to Normal Brain Tissues

**Figure 2:**
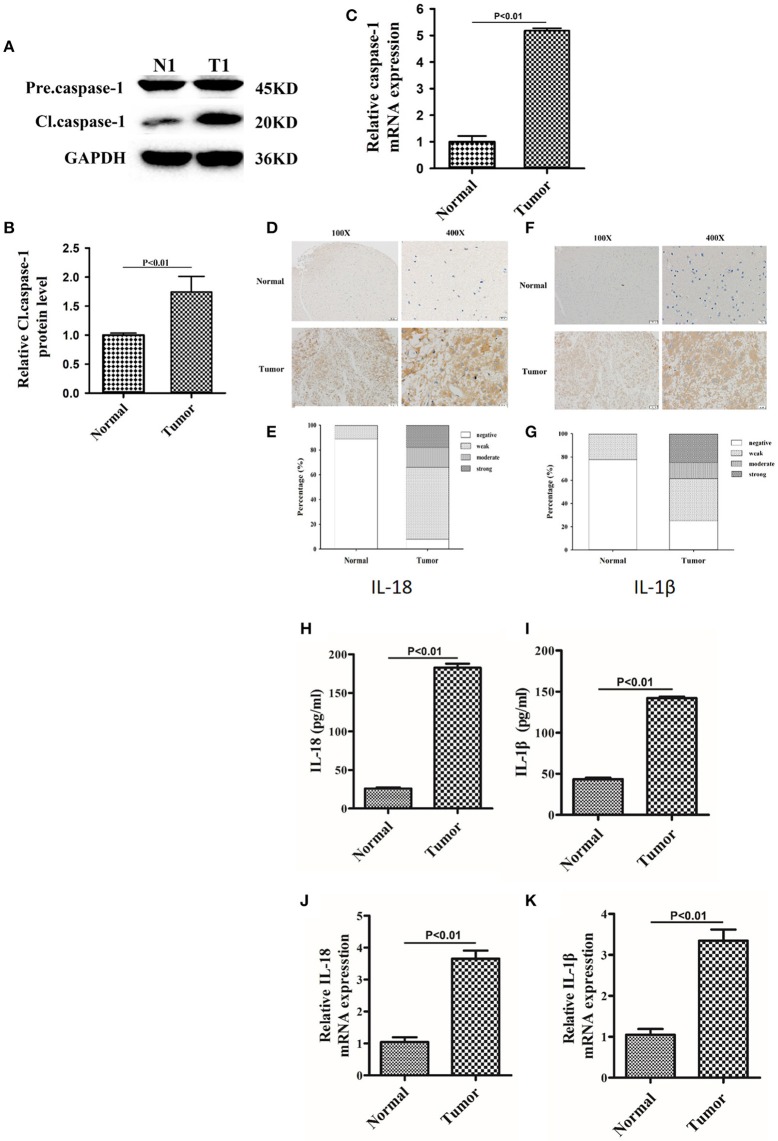
The expression of caspase-1, IL-18, and IL-1β were significantly upregulated in glioma than normal brain tissues. **(A)** Protein levels of caspase-1 and cleaved caspase-1 in normal brain tissues and gliomas. **(B)** Protein levels of cleaved caspase-1 was quantified by densitometric analysis using NIH ImageJ, normalized to β-actin and presented as fold changes compared to the first lane (arbitrarily set at a value of 1). **(C)** mRNA levels of caspase-1 between normal brain tissues and gliomas were compared by qPCR. Unpaired student *t*-test was applied for statistical analysis, *p* < 0.05 was considered as statistically significant. **(D,F)** IHC staining of IL-1β and IL-18 in normal brain tissues and gliomas. The pictures were captured using both 100 × and 400 × objectives. **(E,G)** Statistical analysis were performed to demonstrate whether the expression levels of IL-18 and IL-1β in normal brain tissues and gliomas are correlated with histological grades. **(H,I)** Tissue homogenates of normal brain tissue and gliomas was collected and assayed for IL-1β and IL-18 using human ELISA kits. **(J,K )** mRNA levels of IL-1β and IL-18 between normal brain tissues and gliomas were compared by qPCR. Unpaired student *t*-test was applied for the statistical analysis, *p* < 0.05 was considered as statistically significant.

Since berberine is able to inhibit inflammation both *in vitro* and *in vivo*, we first determined the immunogenicity of the gliomas obtained from our center. Caspase-1 is a cysteine protease that activates proinflammatory cytokines IL-18 and IL-1β; thus, we first determined the gene expression and protein level of caspase 1 in clinical samples (15). As demonstrated in Figures 2A,B, the protein expression of cleaved caspase-1 (activated form) was significantly increased in glioma tissues as compared to normal brain tissues. Furthermore, we demonstrated that mRNA expression of caspase-1 was also significantly upregulated in glioma tissues compared to normal brain tissues, suggesting that transcriptional upregulation contributes to increased protein expression of cleaved caspase-1 (Figure 2C). Immunohistochemical analysis showed that the expression of IL-18 and IL-1β were significantly upregulated in glioma tissues compared to normal brain tissues (Figures 2D–G). These findings were further confirmed by ELISA. Figures 2H,I show that the protein concentration of IL-18 was 8 times higher in gliomas as compared to normal brain tissues, while the protein concentration of IL-1β was 3 times higher. Mechanistically, the increased protein expressions of IL-18 and IL-1β may also be explained by transcriptional regulation because mRNA levels of IL-18 and IL-1β were significantly increased (Figures 2J,K).

### Caspase1 Inactivation Significantly Decreases IL-18 and IL-1β Proteins and Leads to U87 Cell Death

**Figure 3:**
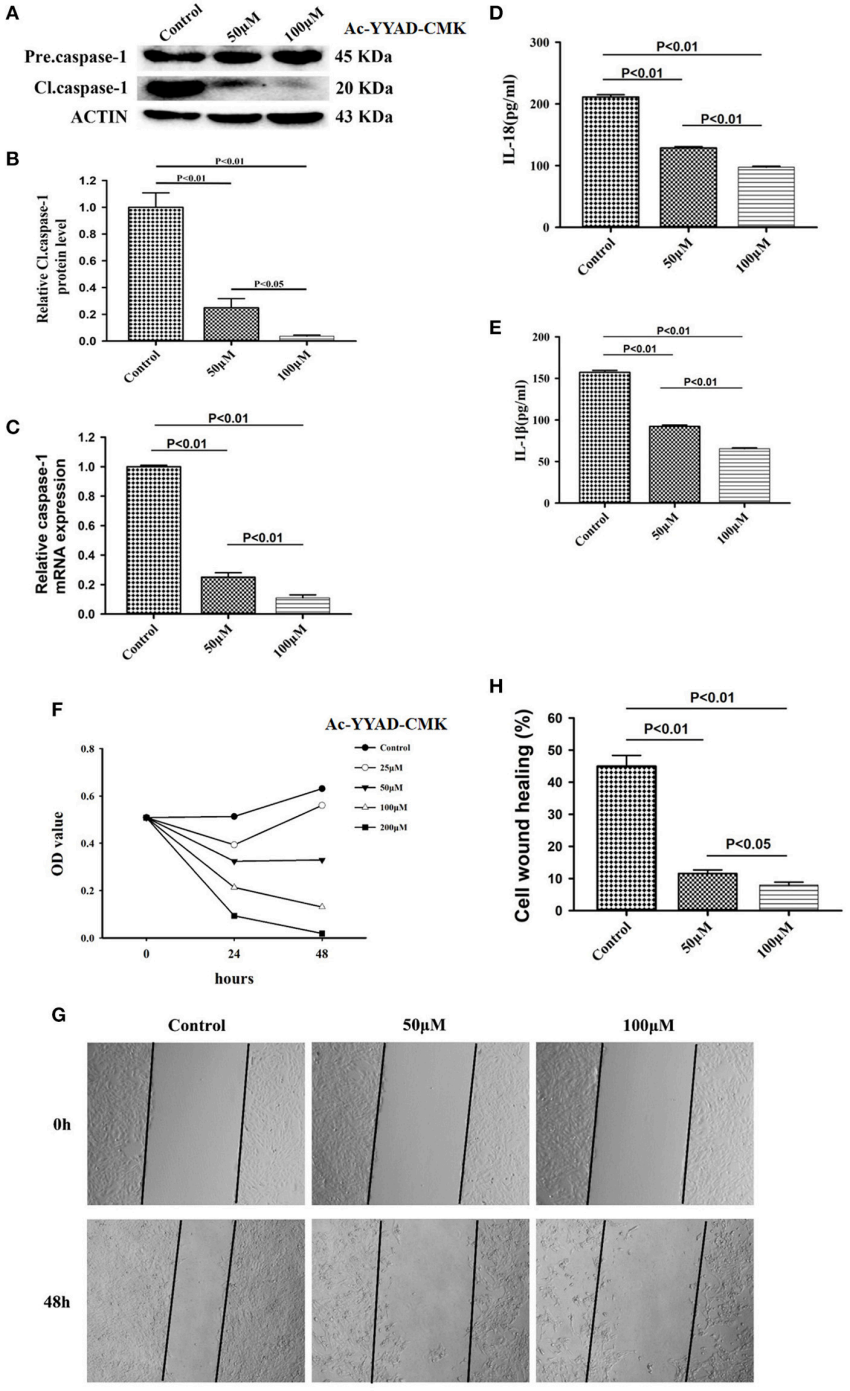
Caspase1 inactivation significantly decreased the protein expression of IL-18 and IL-1β and led to U87 cell death. **(A)** U87 cells were either sham-treated or with Ac-YYAD-CMK at 50 and 100 μM for 24 h. The cell lysates were collected and processed for western blotting of caspase-1, and β-actin was used as a loading control. **(B)** Protein levels of cleaved caspase-1 was quantified by densitometric analysis using NIH ImageJ as described. **(C)** mRNA was extracted from U87 cells treated with either 50 or 100 μM Ac-YYAD-CMK. mRNA levels of caspase-1 was analyzed by qPCR as described. **(D,E)** Cell supernatants of U87 cells treated with either 50 or 100 μM Ac-YYAD-CMK was collected and assayed for IL-1β and IL-18 using human ELISA kits. **(F)** Cell viability was determined by the MTT assay at multiple time points after Ac-YYAD-CMK treatment. **(G,H)** Cell migration was examined using the wound scratch assay in U87 cells treated with caspase-1 inhibitor at different dosages as indicated for 48 h. Cell migration was quantified as the reduction of the wound square as described in the section Materials and Methods.

Caspase-1 belongs to a prototypical member of the inflammatory caspases and is responsible for the maturation of pro-interleukin IL-1β and pro-IL-18 (15). Downstream of caspase-1, production of IL-1β and IL-18 is highly associated with the status of caspase-1. We first performed a dose-dependent experiment to determine the optimal working concentration of caspase-1 inhibitor, Ac-YYAD-CMK. We found that 50 and 100 μM Ac-YYAD-CMK treatment significantly decreased the protein expression of cleaved caspase-1 (Figures 3A,B), suggesting that the activation of caspase-1 is attenuated. Moreover, we found that the mRNA level of caspase-1 is also significantly downregulated, indicating that the effect is due to transcriptional regulation (Figure 3C). Further, we found that Ac-YYAD-CMK significantly attenuated the release of IL-1β and IL-18 as detected by ELISA, suggesting that the production of IL-1β and IL-18 are regulated by caspase-1 activity (Figures 3D,E). Since our clinical study confirmed that glioma samples have an upregulated protein expression of IL-1β and IL-18, we questioned whether inhibition of the production of IL-1β and IL-18 leads to glioma cell death. We therefore performed the MTT assay to evaluate the potential cytotoxic effect of Ac-YYAD-CMK on U87 cell death. We found that the application of Ac-YYAD-CMK significantly resulted in a profound cell death of U87 cells in a dose-dependent manner (Figure 3F). Furthermore, wound healing experiments showed that Ac-YYAD-CMK significantly attenuated the migration ability of U87 cells, in which decreased closure rates were observed in the wound healing assay (Figures 3G,H).

### Berberine Exhibit Inhibitory Effects on Caspase-1, IL-18, and IL-1β Proteins

**Figure 4:**
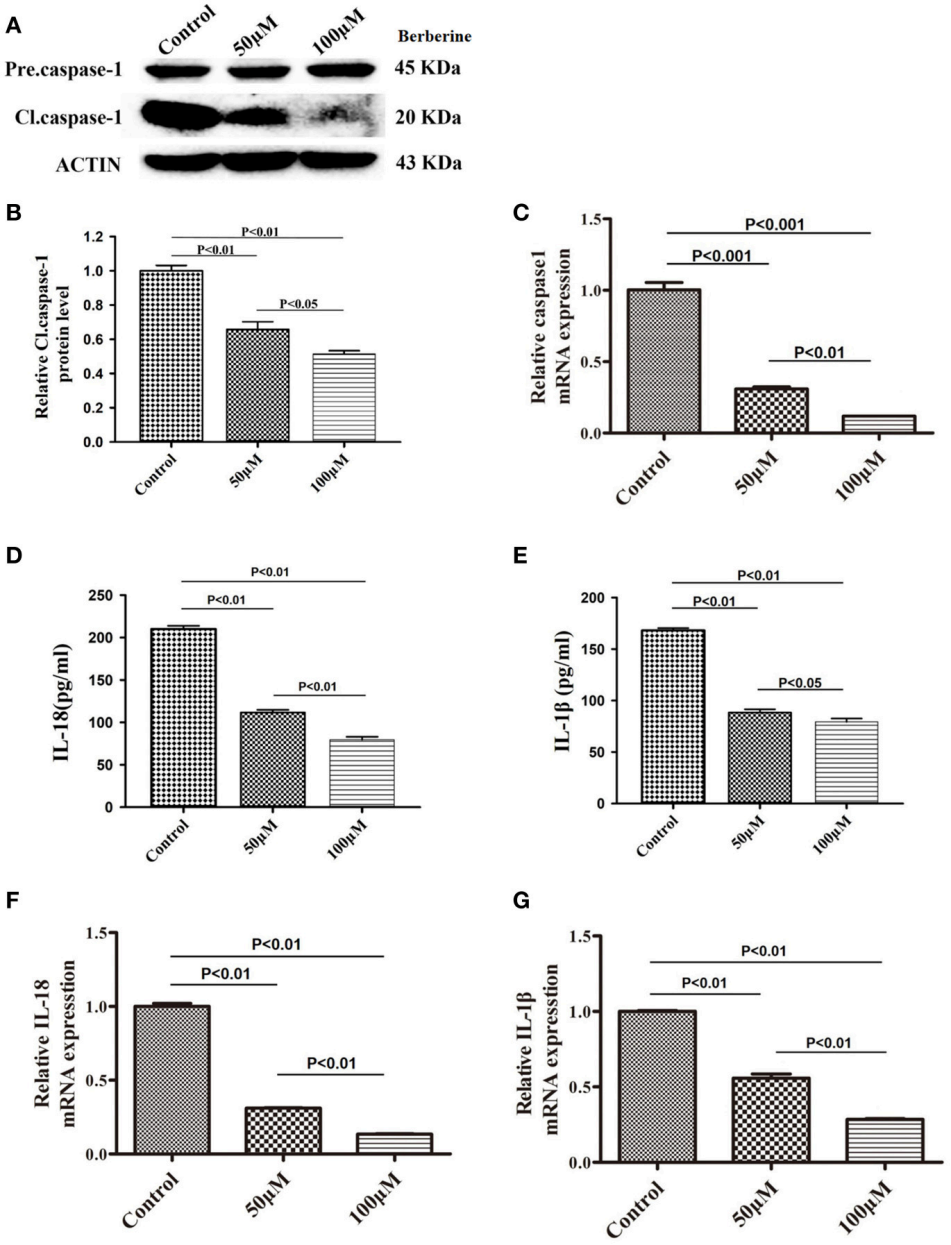
Inhibitory effects of berberine on protein expression of caspase-1, IL-18 and IL-1β. **(A)** U87 cells were either sham-treated or with berberine at 50 and 100 μM for 24 h. The cell lysates were collected and processed for western blotting of caspase-1, and β-actin was used as a loading control. **(B)** Protein levels of cleaved caspase-1 was quantified by densitometric analysis using NIH ImageJ as described. **(C)** mRNA was extracted from U87 cells treated with either 50 or 100 μM berberine. mRNA levels of caspase-1 was analyzed by qPCR as described. **(D,E)** Cell supernatants of U87 cells treated with either 50 or 100 μM berberine was collected and assayed for IL-1β and IL-18 using human ELISA kits. **(F,G)** mRNA was extracted from U87 cells treated with either 50 or 100 μM berberine. mRNA levels of IL-1β and IL-18 were analyzed by qPCR as described.

We next examined if berberine targets IL-18 and IL-1β to mediate its anti-inflammatory effect in U87 cells. We found that protein levels of cleaved caspase-1 were significantly decreased after berberine treatment (Figures 4A,B). QPCR results also demonstrated that berberine treatment led to the downregulation of caspase-1 mRNA, suggesting that berberine may inhibit caspase-1 transcription (Figure 4C). Moreover, berberine treatment resulted in a significant reduction of IL-18 and IL-1β at both the transcriptional and translational levels (Figures 4D-G), suggesting that berberine may regulate the production of IL-18 and IL-1β through inactivation of caspase-1-mediated pathways.

### The Potential of Berberine in Glioma Treatment

**Figure 5:**
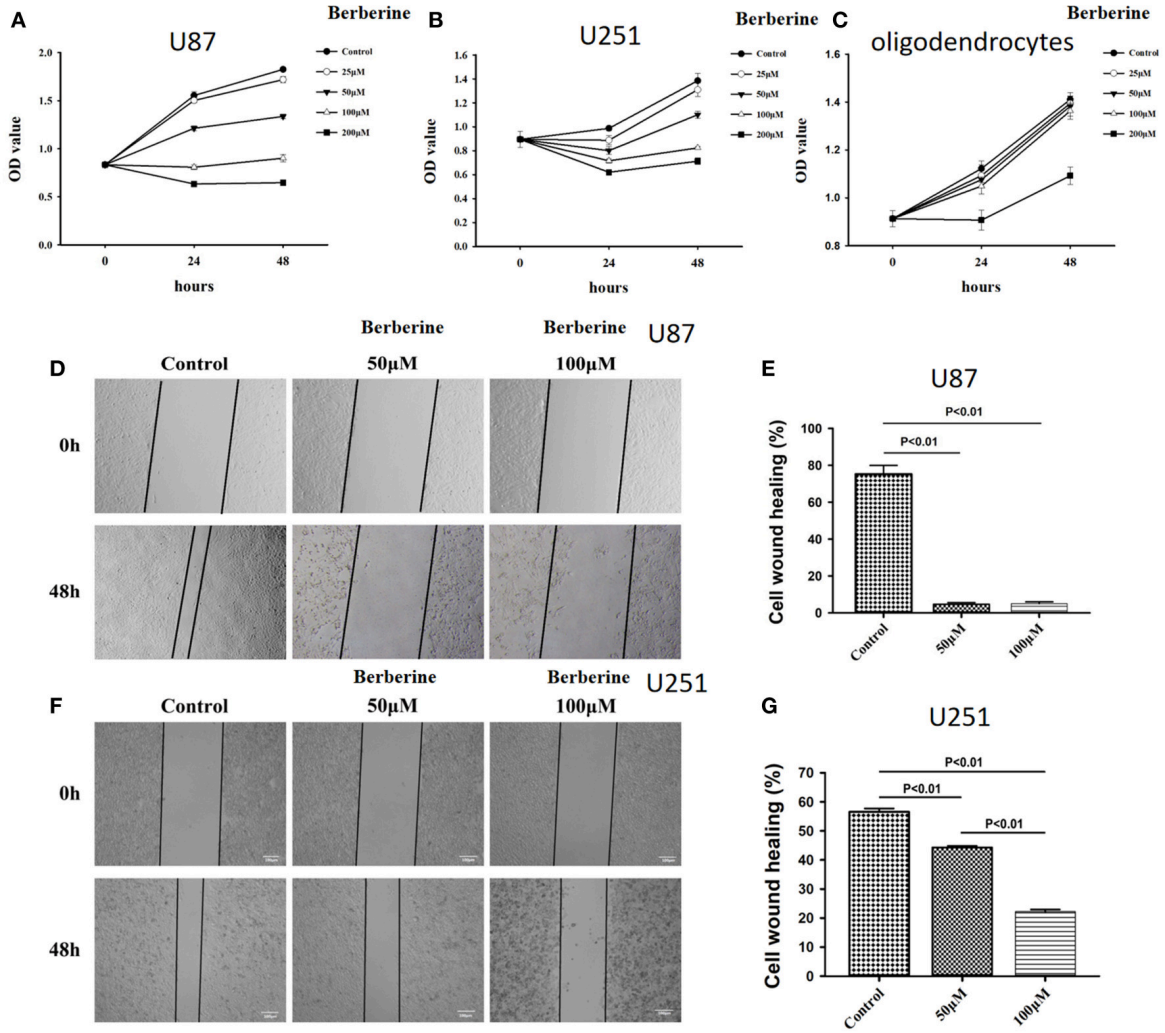
The effects of berberine on cell viability and cell migration. **(A–C)** Cell viability was determined by the MTT assay at multiple time points after berberine treatment **(A)** U87, **(B)** U251, and **(C)** Oligodendrocyte. **(D,F)** Cell migration was examined using the wound scratch assay in U87 and U251 cells treated with berberine at different dosage as indicated for 48 h. **(E,G)** Cell migration was quantified as the reduction of the wound square as described in the section Materials and Methods.

**Figure 6:**
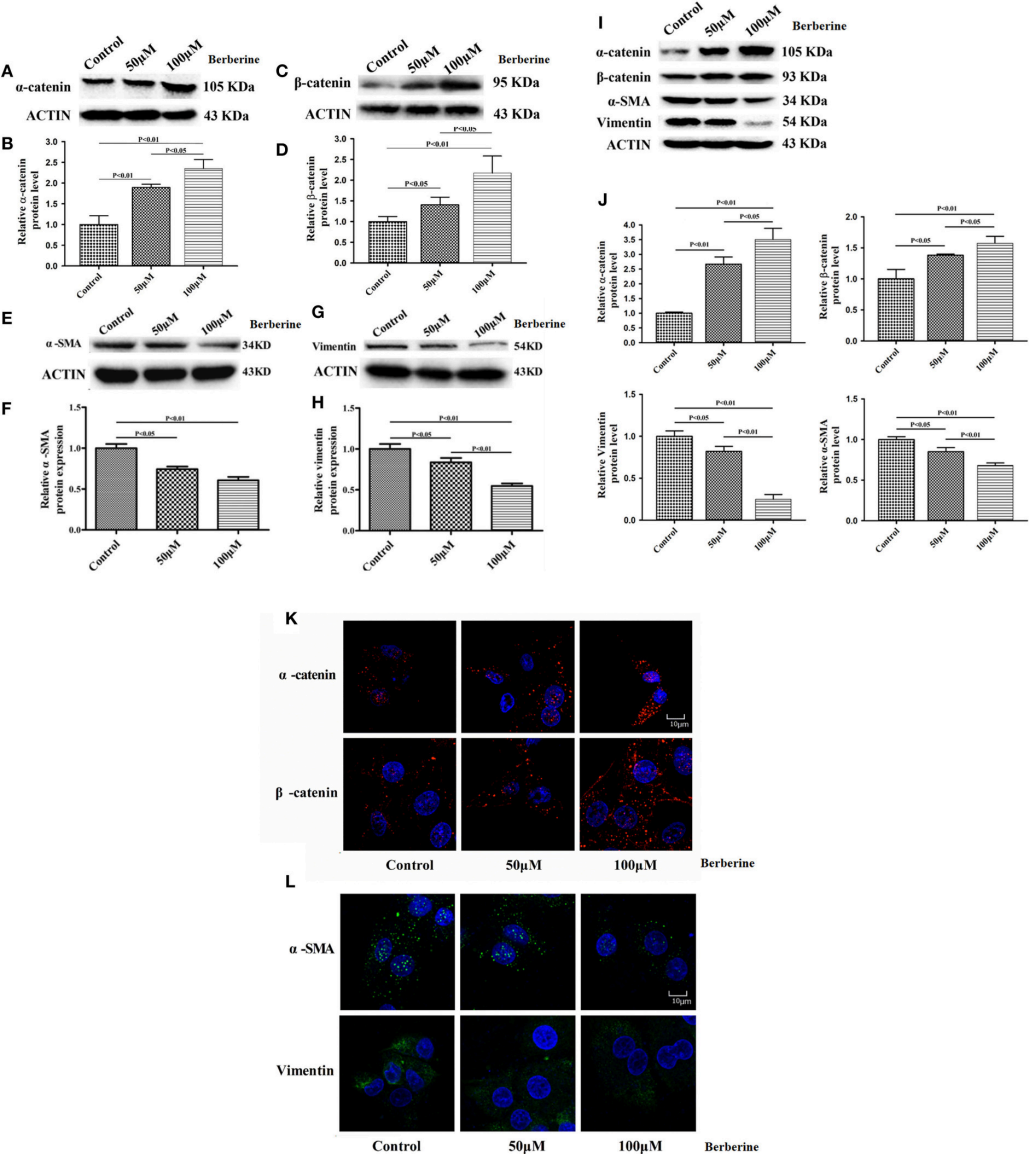
The effects of berberine treatment on EMT process. U87 and U251 cells were either sham-treated or with berberine at 50 and 100 μM for 48 h. The cell lysates were collected and processed for western blotting of β-catenin, α-catenin, vimentin and α-SMA, and β-actin was used as a loading control **(A,C,E,G,I)**. Protein levels of β-catenin, α-catenin, vimentin, and α-SMA were quantified by densitometric analysis using NIH ImageJ as described **(B,D,F,H,J)**. U87 cells were treated with berberine as above and then immunocytochemical staining was conducted using anti-β-catenin (red), anti-α-catenin (red), anti-vimentin (green), and anti-α-SMA antibodies (green). Nuclei were counterstained using DAPI (blue). Scale bars are 10 μM **(K,L)**.

Considering the fact that berberine significantly attenuates the production of IL-18 and IL-1β, which are normally highly expressed in gliomas, we questioned if berberine treatment would significantly decrease the viability of glioma cells. The MTT assay showed that berberine treatment results in a significant reduction of cell viability of U87 and U251 in a dose-dependent manner, but oligodendrocyte viability was not affected (Figures 5A–C). This suggests that berberine has a specific anti-proliferation effect on glioma cells. Next, we performed wound healing assays to evaluate the effects of berberine on glioma migration. Berberine treatment significantly inhibited cell migration of both U87 and U251 cells (Figures 5D,F). Moreover, wound width was significantly increased after either 50 or 100 μM berberine treatment as compared to control group (Figures 5E,G), suggesting that berberine has an inhibitory effect on cell migration. The phenotype transition of epithelial cells into motile mesenchymal cells, a process called epithelial-mesenchymal transition (EMT), is integral in all aspects of cell biology, including cell development, wound healing, and stem cell behavior. In addition, EMT contributes pathologically to fibrosis and cancer progression (16). In cancer biology, it is well accepted that EMT is involved in the generation of invasive cells and acquisition of cancer stem cell properties. Previous reports have demonstrated that berberine can reverse the EMT process in multiple cancer types including cervical cancer, prostate cancer, lung cancer and melanoma (17–20). We therefore evaluated the effects of berberine on glioma EMT. Using western blots, we showed that berberine treatment significantly increased the protein expression of β-catenin and α-catenin, two markers of the epithelium, and decreased the protein expression of vimentin and α-SMA, two mesenchymal markers in both U87 and U251 cells (Figures 6A–J). Similarly, we found that ~80% of U87 cells treated with berberine exhibited increased staining of β-catenin and decreased staining of vimentin (Figures 6K,L). Taken together, these results suggest that berberine can inhibit the process of EMT.

### Berberine Inhibits Caspase-1 Activation via ERK1/2 Signaling Pathway

**Figure 7:**
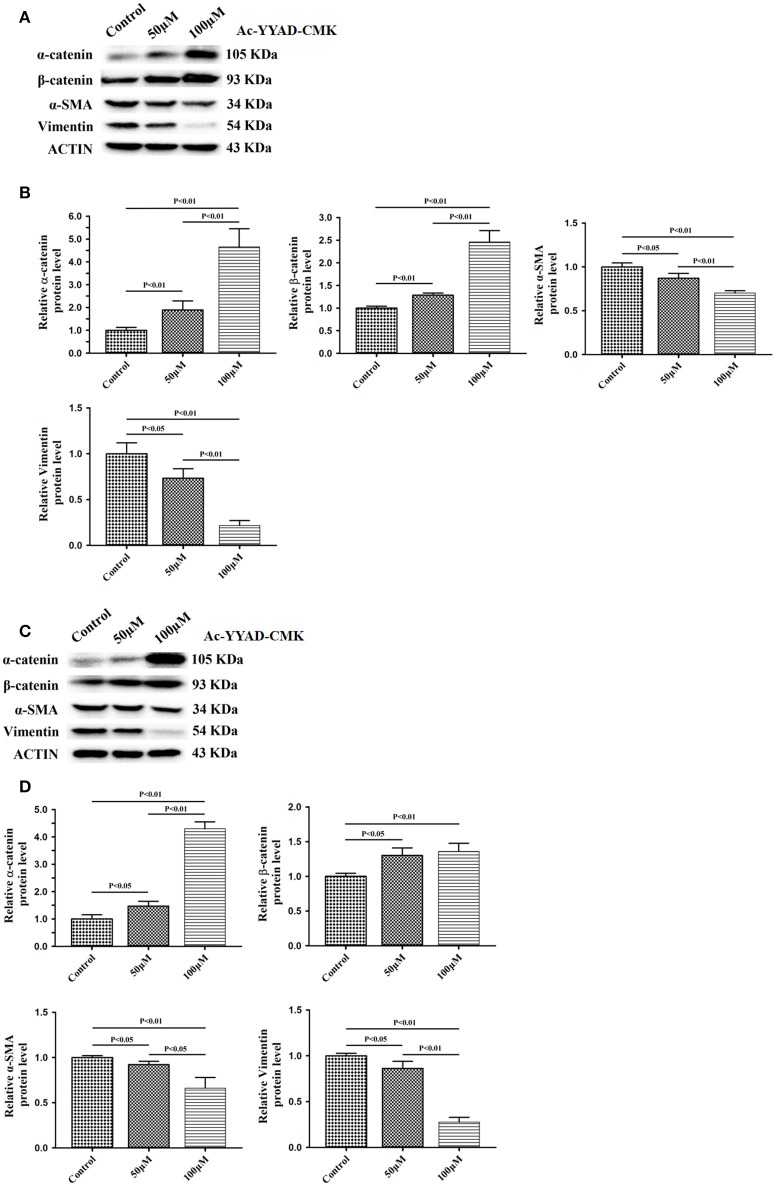
Caspase-1 inactivation prevents EMT in U87 and U251 cells. U87 and U251 cells were either sham-treated or with Ac-YYAD-CMK at 50 and 100 μM for 48 h. The cell lysates were collected and processed for western blotting of β-catenin, α-catenin, vimentin and α-SMA, and β-actin was used as a loading control **(A,C)**. Protein levels of β-catenin, α-catenin, vimentin, and α-SMA were quantified by densitometric analysis using NIH ImageJ as described **(B,D)**.

**Figure 8:**
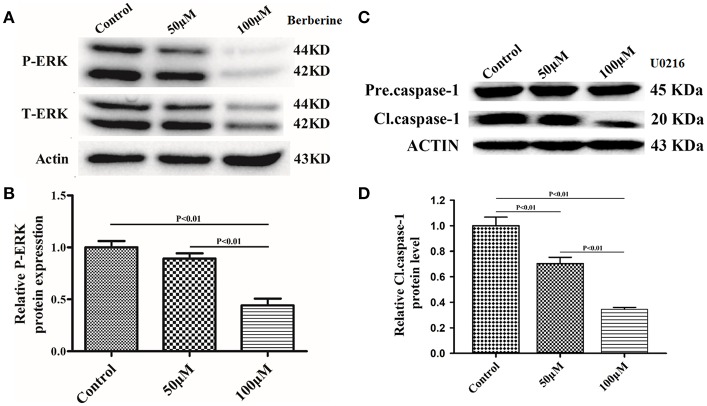
Berberine inhibits caspase-1 activation via the ERK1/2 signaling pathway. **(A,C)** The cell lysates were collected and processed for western blotting of p-ERK and caspase-1, and β-actin was used as a loading control. **(B,D)** Protein levels of p-ERK and caspase-1 were quantified by densitometric analysis using NIH ImageJ as described.

Next, we used Ac-YYAD-CMK to determine if the inhibitory effect of berberine on EMT is dependent on caspase-1-mediated pathways. Both U87 and U251 cells were treated with Ac-YYAD-CMK and EMT markers were examined as described above. Inhibiting caspase-1 with Ac-YYAD-CMK significantly increased the protein expression of β-catenin and α-catenin, two markers of epithelium, and decreased the protein expression of vimentin and α-SMA, two mesenchymal markers in both U87 and U251 cells (Figures 7A–D), suggesting that berberine inhibits EMT via caspase-1-mediated pathways. It has been reported that berberine induces senescence of human glioma cells by downregulating the extracellular kinase/mitogen-activated protein kinase (ERK/MAPK) signaling pathway (21). As a major mediator of cell invasion and proliferation, aberrant ERK1/2 activation has been shown to be responsible for tumor progression (22). We therefore questioned whether the inhibitory effect of berberine on cell viability is mediated by ERK1/2 signaling. Our results showed that berberine treatment on U87 cells significantly decreased the activation of ERK1/2 in a dose-dependent manner (Figures 8A,B). Moreover, we found that inhibiting ERK1/2 signaling using U0216 decreased the protein expression of cleaved caspase-1, suggesting that berberine may regulate caspase-1 activation via ERK1/2 signaling (Figures 8C,D). To further exclude the possibility that berberine halts other signaling pathways that contribute to the inactivation of caspase-1, we examined the effect of berberine on the activation of JNK, p-38 and PI3K pathways. Levels of p-JNK, p-HSP27 and p-Akt did not significantly change after berberine treatment, indicating that neither JNK, p38 nor PI3K pathways participate in the inactivation of caspase-1 (Data not shown). Taken together, our results suggest that berberine may seves as a modulator of ERK1/2 signaling, responsible for both tumor cell death and inactivation of caspase-1-mediated signaling.

## Discussion

Berberine is a natural botanical alkaloid that is found in the roots and bark of the Berberis species (9). Although the exact mechanisms remain unknown, berberine possesses a variety of biological functions, including anti-diarrheal, anti-fungal, anti-diabetic, hepatoprotective, and cardioprotective effects (23). Here, we report that berberine may serve as a potent antitumor agent in glioma therapy. It is well-established that EMT in glioma cells can lead to their acquisition of invasive and metastatic properties (24, 25). In the current study, we found that berberine significantly increased the expression of β-catenin and decreased the expression of vimentin in U87 and U251 cells. As a result, berberine treatment significantly attenuated cell migration rate, a marker of cancer metastasis. These results together demonstrate an antitumor property of berberine and its clinical potential as an inhibitor of metastasis in glioma cells.

The inflammatory response is a key component of the tumor microenvironment, which is mainly orchestrated by immune cells that are indispensable for tumor proliferation, survival and migration. IL-1β a potent multifunctional pro-inflammatory polypeptide, is usually produced by monocytes and tissue macrophages (26). Numerous reports have indicated that IL-1β is produced during inflammation, and this cytokine stimulates tumor cell proliferation and promotes angiogenesis and tumor invasion (27). Here, we first demonstrated the stimulatory effect of IL-1β and IL-18 on the migration and invasion of U87 cells. These pro-tumoral effects were significantly inhibited by Ac-YYAD-CMK and berberine. Furthermore, it has been reported that IL-1β promotes glioma migration, invasion, and proliferation with synchronous elevation of MMP-2 and MMP-9 (28). IL-1β is a dominant molecule in the hierarchical cytokine signaling cascade in the CNS. Activation of IL-1β-mediated NF-κB leads to the cytoplasmic release and nuclear translocation of NF-κB, which, in turn, controls the expression of inflammatory and oncogenic genes (29). On the other hand, it was reported that berberine can inhibit IKK activation, an upstream modulator of the NF-κB pathway. As a result, berberine treatment causes apoptosis of primary effusion lymphoma cells (29). Given the importance of IL-1β-mediated intracellular signaling, our study first focused on berberine as a key modulator of tumor microenvironment by targeting IL-1β. We speculated that downstream of IL-1β, NF-κB signaling is likely to be responsible for glioma metastasis and tumor EMT as target genes of NF-κB, such as MMPs and vascular endothelial growth factors, are highly associated with tumor invasion. Similar findings have also been observed in lymphoma. The study found that berberine can inhibit IKK activation and causes efficient apoptosis of primary effusion lymphoma cells. Apart from the NF-κB pathway, our study also demonstrated the effects of berberine on ERK1/2 signaling, which regulates cell proliferation. Targeting the ERK1/2 pathway is a major strategy in a variety of cancer therapeutics. In our study, we found that berberine can significantly inhibit ERK1/2 activation in U87 cells and lead to U87 cell death.

Despite the extensive insights gained in the therapeutic targets in gliomas, there have been no significant benefits obtained from employing such therapies. Our study on berberine has potentially revealed a novel strategy for glioma treatment. Further studies will be conducted to understand the mechanisms by which berberine hampers tumor invasion and the EMT process. Elucidation of pharmacological diversity of berberine is likely to further establish a solid foundation for its application for cancer treatment.

## Ethics Statement

This study was carried out in accordance with the recommendations of the ethics committee of Harbin Medical University with written informed consent from all subjects. All subjects gave written informed consent in accordance with the Declaration of Helsinki. The protocol was approved by the ethics committee of Harbin Medical University.

## Author Contributions

LT, HQ, and HD designed the studies. CX, YW, YQ, HL, YZ, TX, and HQ performed the experiments. HD, XQ and LT wrote and revised the manuscript.

### Conflict of Interest Statement

The authors declare that the research was conducted in the absence of any commercial or financial relationships that could be construed as a potential conflict of interest.

## Abbreviations

GBM: glioblastoma multiforme
OS: overall survival
IL-1: interleukin-1
IL-1R: interleukin-1 receptor
CGGA: Chinese Glioma Genome Atlas network
TCGA: The Cancer Genome Atlas
REMBRANDT: Repository for Molecular Brain Neoplasia
EMT: epithelial–mesenchymal transition
ERK/MAPK: extracellular signal-regulated kinase/mitogen-activated protein kinase
α-SMA: α-smooth muscle actin
MMP: matrix metalloproteinase
NF-κB: nuclear factor kappa B
DMSO: dimethyl sulfoxide
ELISA: Enzyme-linked immunosorbent assay
PVDF: polyvinylidene difluoride
SEM: standard error of mean
ANOVA: analysis of variance
JNK: c-Jun N-terminal kinase
HSP: heat shock protein.
